# A local-optimization refinement algorithm in single particle analysis for macromolecular complex with multiple rigid modules

**DOI:** 10.1007/s13238-015-0229-2

**Published:** 2015-12-17

**Authors:** Hong Shan, Zihao Wang, Fa Zhang, Yong Xiong, Chang-Cheng Yin, Fei Sun

**Affiliations:** Department of Biophysics, College of Basic Medical Sciences, Peking University Health Science Center, Beijing, 100191 China; National Laboratory of Biomacromolecules, Institute of Biophysics, Chinese Academy of Sciences, Beijing, 100101 China; Key Lab of Intelligent Information Processing and Advanced Computing Research Lab, Institute of Computing Technology, Chinese Academy of Sciences, Beijing, 100190 China; University of Chinese Academy of Sciences, Beijing, 100049 China; Department of Molecular Biophysics and Biochemistry, Yale University, New Haven, CT 06511 USA; Center for Biological Imaging, Institute of Biophysics, Chinese Academy of Sciences, Beijing, 100101 China

**Keywords:** cryo-electron microscopy, single particle analysis, conformational heterogeneity, rigid module, local optimization refinement

## Abstract

Single particle analysis, which can be regarded as an average of signals from thousands or even millions of particle projections, is an efficient method to study the three-dimensional structures of biological macromolecules. An intrinsic assumption in single particle analysis is that all the analyzed particles must have identical composition and conformation. Thus specimen heterogeneity in either composition or conformation has raised great challenges for high-resolution analysis. For particles with multiple conformations, inaccurate alignments and orientation parameters will yield an averaged map with diminished resolution and smeared density. Besides extensive classification approaches, here based on the assumption that the macromolecular complex is made up of multiple rigid modules whose relative orientations and positions are in slight fluctuation around equilibriums, we propose a new method called as local optimization refinement to address this conformational heterogeneity for an improved resolution. The key idea is to optimize the orientation and shift parameters of each rigid module and then reconstruct their three-dimensional structures individually. Using simulated data of 80S/70S ribosomes with relative fluctuations between the large (60S/50S) and the small (40S/30S) subunits, we tested this algorithm and found that the resolutions of both subunits are significantly improved. Our method provides a proof-of-principle solution for high-resolution single particle analysis of macromolecular complexes with dynamic conformations.

## INTRODUCTION

Single-particle analysis (SPA) of electron cryo-microscopy (cryo-EM) has become an efficient method to reveal structural information of macromolecular complexes. In theory, it is possible to solve a 3 Å resolution structure when thousands of single particle images are averaged (Henderson, 1995). Nowadays, with improved detectors and image processing techniques, the prediction comes true with not only large, highly symmetrical viruses (Wang et al., 2014; Zhang et al., 2010) or asymmetrical ribosome (Fischer et al., 2015), but also small membrane proteins (Liao et al., 2013) (TRPV1), whose structures were resolved at near-atomic resolutions.

Besides the rapid progress in pushing resolution, however, intrinsic sample heterogeneity in composition or conformation is becoming a threshold stopping us obtaining higher-resolution structure. The current solution to deal with both heterogeneity problems is to divide the data set into different classes with each class corresponding to one homogenous composition/conformation (Leschziner and Nogales, 2007). A couple of classification methods have been developed, such as the normal mode analysis (NMA) method that uses simulated models as references for multi-reference supervised classification (Brink et al., 2004; Jin et al., 2014), 3D multivariate statistical analysis (MSA) that projects a 3D mask of the area with the most variance to a series of 2D images in the same orientation and performs classification focusing on the masked highly varied regions (Penczek et al., 2006a; Penczek et al., 2006b; Zhang et al., 2008), and a Bayesian based 3D classification method (Scheres, 2012b). These classification methods can work well upon the assumption that the heterogeneous sample only contains a finite number of compositions/conformations.

In practice, for many macromolecular complexes that exhibit heterogeneity with dynamic conformations and thereby an infinite conformational states, the above classification methods may not produce a good result. A typical scenario is that the macromolecular complex comprises a number of stable modules (such as domains, subunits, or sub-complexes) that can be treated as rigid bodies, the overall flexibility of the complex is due to the dynamic slight fluctuations of the relative orientations and positions between rigid modules. In these cases, the conventional SPA approach will yield a 3D reconstruction with smeared densities (i.e. a decreased resolution) because SPA approach assumes that all the particles within a class have an identical structure while this is not correct for such flexible complexes and the assigned parameters could be inaccurate for all of the modules. In addition, conventional classification approaches are also not able to classify the infinite continuous conformations of the complex into a finite number of discrete states with enough homogeneity within a class. Examples for these kinds of macromolecular complexes include the ribosome containing two subunits with relative motion (Bai et al., 2013) and the splicesome with substantial flexibility among subunits that is still poorly resolved by cryo-EM SPA approach (Azubel et al., 2004). Besides developing new sample preparation and freezing procedures to reduce the flexibility and heterogeneity, there is a great need of new image-processing algorithms to adequately treat the dynamic conformation problem.

Here we report a new image-processing algorithm that can yield a better resolution by resolving the accurate orientation and shift parameters of each individual structural module respectively. Since the orientation and shift parameters of each module are searched within a local range and only the local area of the particle image is counted, we call this method as the local optimization refinement algorithm (LO-refinement). In a test case, we used the ribosome (80S or 70S) that has two rigid modules (60S/50S or 40S/30S) with the fluctuating relative orientations and positions to prove the concept of this method.

## THEORY AND ALGORITHM

The LO-refinement is based on the assumption that the imaged macromolecular complex comprises a number of rigid modules with slightly varying relative orientations and positions between different modules. The relative orientation and position between any two modules can fluctuate due to module rotation and shift. The goal of the LO-refinement is to resolve a higher resolution structure of each rigid module.

**Figure 1:**
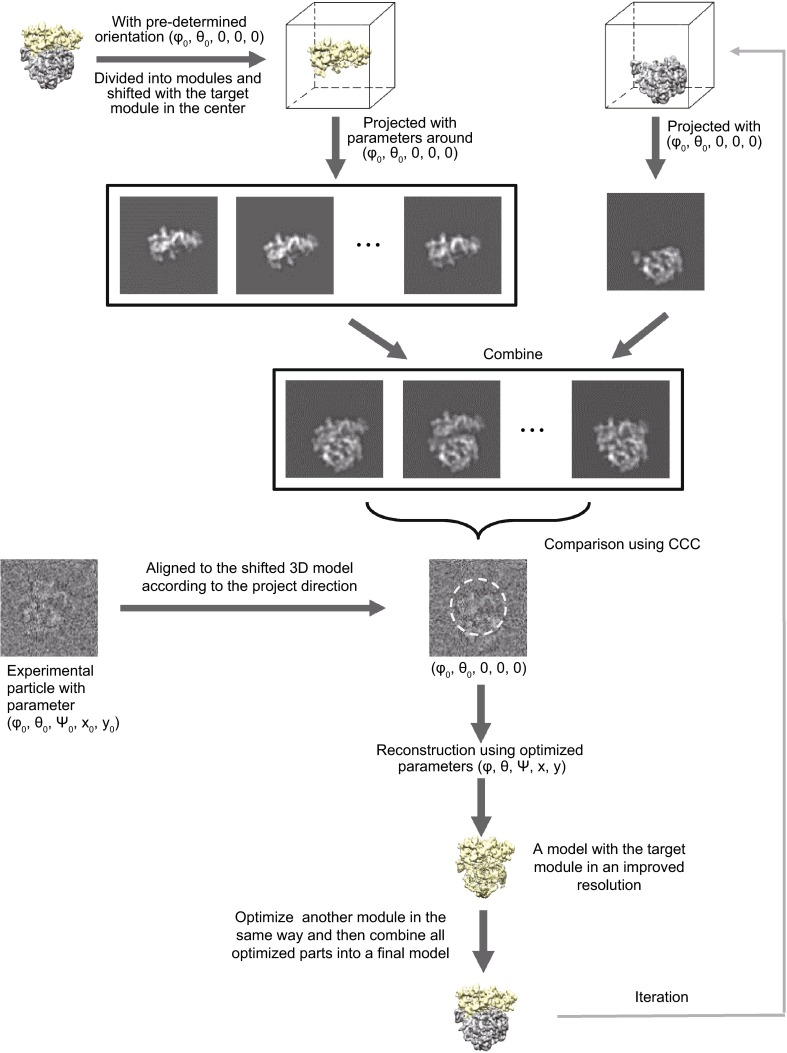
Schematic illustration of the LO-refinement method. The model reconstructed from the last iteration of conventional SPA is split into modules. Then the model is shifted with the target module in the center. Thereafter, the target module is projected with the parameters around the preliminary determined ones while the other module is projected with the preliminary determined parameters. Then the projections are combined into a set of simulated projections. A comparison using cross correlation coefficient (CCC) between the simulated and experiment projections is performed and only the region inside the mask of the target module is counted. The refined parameters of the target module are determined with the highest CCC. A new reconstruction is performed using these newly refined parameters. The same procedure is performed for the other modules and all the refined modules are combined together to yield an update model for the next iteration

In initial step, we use conventional SPA routine to process the raw images of particles and obtain a refined 3D map and full set of preliminary alignment parameters. The resolution of the refined map is restrained due to the inaccuracy of the alignment that is caused by the heterogeneity of the particles. Then we separate the refined map into different modules according to prior knowledge under the assumption that the resolution of the refined map has been high enough to discriminate different modules. Starting from the preliminary alignment parameters, we focus on a single rigid module, optimize its orientation and position and thereafter compute a new reconstruction with the refined parameters. Since the orientation and position of the target module is now determined more accurately, the resolution of the final reconstructed map is improved for that module but likely decreased for other modules due to the even lower accuracy of their parameters. By applying the same operation procedure to each individual module, the resolutions of all the reconstructed modules could be improved. To optimize the orientation of each individual module, we consider all the possible positions and orientations of the target module within a local range that is caused by conformational dynamics in 3D space and then search the optimized parameters by maximizing the cross-correlation coefficient (CCC) between the projections of the 3D models and the raw particle image. The main conception of the method is shown in Fig. 1.

### The rationality of using cross-correlation coefficient

The cross-correlation coefficient (CCC) of two images ${{f}}_{1} \left( {{{r}}_{{{j}}} } \right)$ and ${{f}}_{2} \left( {{{r}}_{{{j}}} } \right)$ is defined as (Frank, 1996):

1 $$ \rho_{12} = \frac{{\mathop \sum \nolimits_{j = 1}^{J} \left[ {f_{1} \left( {r_{j} } \right) - {<}f_{1}\!\!{>}}\!\!\right]\left[ {f_{2} \left( {r_{j} } \right) - {<}f_{2}\!\!{>}}\!\! \right]}}{{\left\{ {\mathop \sum \nolimits_{j = 1}^{J} [f_{1} \left( {r_{j} } \right) - {<}f_{1}\!\!{>}\!\!]^{2} \mathop \sum \nolimits_{j = 1}^{J} [f_{2} \left( {r_{j} } \right) - {<}f_{2}\!\!{>}\!\!]^{2} } \right\}^{1/2} }} $$

Where, $f\left( {r_{j} } \right)$ is the value of the j-th pixel in the image of *J* dimension, ${<}f_{i}\!\!{>}= 1/J\mathop \sum \limits_{j = 1}^{J} \, f_{i} \left( {r_{j} } \right)$; i = 1,2. This formula is composed of a numerator representing the similarity between two images and a denominator for normalization, resulting $- 1 \le \rho_{12} \le 1$.

Assuming that a molecular complex comprises two modules A and B, the projection of the whole complex $f\left( {{r}_{{j}} } \right)$ is the summation of the projections of two modules $f_{A} \left( {{r}_{{j}} } \right)$ and $f_{B} \left( {{r}_{{j}} } \right)$.

2 $$ f\left( {{r}_{{j}} } \right) = f_{A} \left( {{r}_{{j}} } \right) + f_{B} \left( {{r}_{{j}} } \right) $$

To optimize the orientation of module A, for every experimental particle image $f_{1} \left( {{r}_{{j}} } \right)$, we have

3 $$ f_{1} \left( {{r}_{{j}} } \right) = f_{1A} \left( {{r}_{{j}} } \right) + f_{1B} \left( {{r}_{{j}} } \right) $$

At the same time, we can generate a series of simulated model by setting every possible position and orientation of module A within a local region around its preliminary parameters and get the simulated projections $f_{2} \left( {{r}_{{j}} } \right)$, we have

4 $$ f_{2} \left( {{r}_{{j}} } \right) = f_{2A} \left( {{r}_{{j}} } \right) + f_{2B} \left( {{r}_{{j}} } \right) $$

Where, $f_{2A} \left( {{r}_{{j}} } \right)$ is the projection of module A with adjusted position and orientation and $f_{2B} \left( {{r}_{{j}} } \right)$ is the projection of module B with its preliminarily determined parameters of orientation and shift.

The cross-correlation coefficient $\rho_{12}$ between the simulated projection $f_{2} \left( {{r}_{{j}} } \right)$ and corresponding experimental particle image $f_{1} \left( {{r}_{{j}} } \right)$ can be computed by using formula (1), and the numerator of $\rho_{12}$ can be written as

5 $$ \mathop \sum \limits_{j = 1}^{J} \left[ {f_{1A} \left({r_{j} } \right) - {<}f_{1A}\!\!{>}}\!\! \right]\left[ {f_{2A} \left( {r_{j} } \right) - {<}f_{2A}\!\!{>}}\!\! \right] $$

6 $$ + \mathop \sum \limits_{j = 1}^{J} \left[ {f_{1A} \left( {r_{j} } \right) - {<}f_{1A}\!\!{>}}\!\! \right]\left[ {f_{2B} \left( {r_{j} } \right) - {<}f_{2B}\!\!{>}}\!\! \right] $$

7 $$ + \mathop \sum \limits_{j = 1}^{J} \left[ {f_{1B} \left( {r_{j} } \right) - {<}f_{1B}\!\!{>}}\!\! \right]\left[ {f_{2B} \left( {r_{j} } \right) - {<}f_{2B}\!\!{>}}\!\! \right] $$

8 $$ + \mathop \sum \limits_{j = 1}^{J} \left[ {f_{1B} \left( {r_{j} } \right) - {<}f_{1B}\!\!{>}}\!\! \right]\left[ {f_{2A} \left( {r_{j} } \right) - {<}f_{2A}\!\!{>}}\!\! \right] $$

Where term (5) represents the cross-correlation of projections between experimental and simulated module A. Searching the optimized parameters of module A is equivalent to maximizing this term. Term (6) represents the cross-correlation of projections between experimental module A and pre-determined module B, which is invariant during optimizing. Term (7) represents the cross-correlation of projections between experimental and pre-determined module B, which is also invariant. Term (8) represents the cross-correlation of projections between experimental module B and simulated module A, which varies during searching the parameters of module A. However, since module A and module B have different structures, shapes, positions and orientations, the variance of Term (8) is small during the optimization of module A, especially when applying an appropriate mask to module A. As a result, maximizing *ρ*_12_ is approximately equivalent to maximizing term (5), which is our target. That is to say, although the information of an individual module can not be explicitly separated from that of other modules within one particle projection, maximizing the cross-correlation coefficient between the experimental and simulated whole projections to search the parameters of the target module can, with most probability, yield more precise parameters for the orientation and position of target module in the experimental projection.

### Search range of the target module orientation and position

For the orientation of a rigid module, there are five parameters to be optimized, three orientation angles φ, θ, ψ and two in-plane translations x, y. Here, φ, θ define the projection direction in 3D space, ψ is the in-plane rotation angle and x, y determine the position of the projection in the plane. In an experimental projection, for molecular complexes with limited module motion, the optimal parameters of the target module should be near the preliminarily determined global ones from conventional SPA procedures, resulting in a constrained space for optimizing those five parameters (φ, θ, ψ, x, y) of the target module.

**Figure 2:**
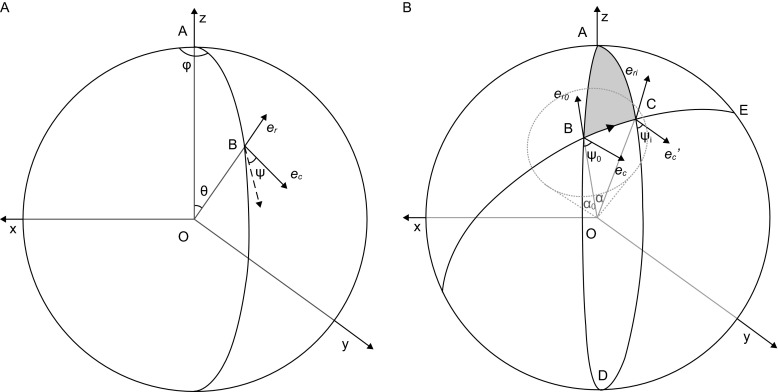
Coordinate system used for searching the optimized orientation of the target module. (A) Definition of the position and orientation of a moving camera in the coordinate system with the object fixed at the origin. The camera plane is assumed perpendicular to the projection direction (OB). Therefore, the position of the camera can be defined by the angle pair (φ, θ) or the spherical unit vector *e*_*r,*_ and its relative in-plane rotation can be defined by the angle ψ between the camera and the meridian (AB). (B) With the preliminary projection direction of OB, the search range of the camera direction is defined by a cone with the semi-angle α_0_ around the preliminary direction OB. When the position of the camera changes from B to C, the direct way for the camera’s moving is along the great arc BC by keeping its angle with the arc unchanged (parallel condition) during move. The difference between the relative in-plane rotation angles ψ_0_ and ψ_i_ is equal to the difference between the angles of ∠DCE and ∠DBE

Here, for convenience, we use the coordinate system with a fixed object at the origin and a moving camera on the surface of a unit sphere (Fig. 2). As shown in Fig. 2A, supposing that the camera plane is always perpendicular to the projection direction, the position of the camera plane is defined by the projection direction with two spherical angles φ and θ. And the in-plane rotation angle ψ of the camera is defined as the angle between the camera and the meridian AB with *e*_*c*_ reflecting the final orientation of the camera. The projection direction (φ, θ) can be represented by the unit vector *e*_*r*_ with the following formula,

9 $$ e_{r} = \left( {\sin \left( {\theta} \right)\cos \left( {\varphi} \right),\sin \left( {\theta} \right)\sin \left( {\varphi} \right),\cos \left( {\theta} \right)} \right) $$

In Fig. 2B, OB is the preliminary projection direction (φ_0_, θ_0_). OC is a projection direction (φ_i_, θ_i_) near OB. For the pre-determined preliminary projection direction *e*_*r0*_ and the projection direction *e*_*ri*_ within the search range, the span angle α by these two directions can be determined with the formula,

10 $$ \alpha = { \arccos }[e_{r0} \cdot e_{ri} ] = \arccos [\sin \left( {{\theta}_{0} } \right)\sin \left( {{\theta}_{i} } \right)\cos \left( {{\varphi}_{0} - {\varphi}_{i} } \right) + \cos \left( {{\theta}_{0} } \right)\cos \left( {{\theta}_{i} } \right)] $$

Thus, the range of optimization search for projection direction (φ_i_, θ_i_) can be confined locally with a pre-defined maximum span angle α_0_ as follows,

11 $$ \left\{ {\left( {{\varphi}_{i} ,{\theta}_{i} } \right)  | {\text{ arccos}}\left[ {\sin \left( {{\theta}_{0} } \right)\sin \left( {{\theta}_{i} } \right)\cos \left( {{\varphi}_{0} - {\varphi}_{i} } \right) + \cos \left( {{\theta}_{0} } \right)\cos \left( {{\theta}_{i} } \right)} \right] \le \alpha_{0} ,\left( {{\varphi}_{i} ,{\theta}_{i} } \right) \in D} \right\} $$

Here, D is a set of evenly distributed projection directions and can be generated by SPIDER command VO NEA.

During the projection direction changed, the apparent in-plane rotation of the camera ψ in the above defined coordinate system will change and the center of the search region of the in-plane rotation angle ψ_i_ should be recomputed at every new projection direction (φ_i_, θ_i_). As shown in Fig. 2B, the way to move camera from B to C with zero in-plane rotation of camera in the coordinate system of camera itself is to keep its angle with the arc BC unchanged during movement. As a result, the apparent in-plane rotation angle ψ_i_ in our defined coordinate system is changed and can be determined with the following relation,

12 $$ {\Psi}_{i} - {\Psi}_{0} { = }\angle DCE - \angle DBC $$

By considering every possible situation on a sphere, we obtain:

When |φ_i_ − φ_0_| ≤π

13 $$ {\Psi}_{i} = {\Psi}_{0} - \, (\pi - \angle B - \angle C)(\varphi_{i} - \varphi_{0} )/|\varphi_{i} - \varphi_{0} | $$

When |φ_i_ − φ_0_| >π

14 $$ {\Psi}_{i} = {\Psi}_{0} { + }(\pi - \angle B - \angle C)(\varphi_{i} - \varphi_{0} )/|\varphi_{i} - \varphi_{0} | $$

Where, ∠B and∠C are the internal angles of spherical triangle Δ ABC that is composed by great circle arcs on a sphere. In case of that A, B and C do not constitute a triangle but locate on a same great circle arc, a simple relationship can be obtained with ψ_i_ = φ_0_ + ψ_0_ − φ_i._ Finally, the search range of ψ for a particular (φ_i_, θ_i_) is

15 $$ \{ {\Psi}_{i} + d_{1} n|n \in Z, - t_{1} \le n \le t_{1} {\text{\} }} $$

Where *d*_1_ is the search step size and *t*_1_ is the number of search steps.

The in-plane shift parameters x and y of the camera are exhaustively searched by

16 $$ \{ d_{2} n|n \in Z, - t_{2} \le n \le t_{2} \} $$

Where *d*_2_ is the search step size and *t*_2_ is the number of search steps.

Over all, we optimize the orientation of the target module by searching all five parameters (φ, θ, ψ, x, y) in a confined range defined by (11), (15) and (16).

### Procedure of local orientation optimization

**Figure 3:**
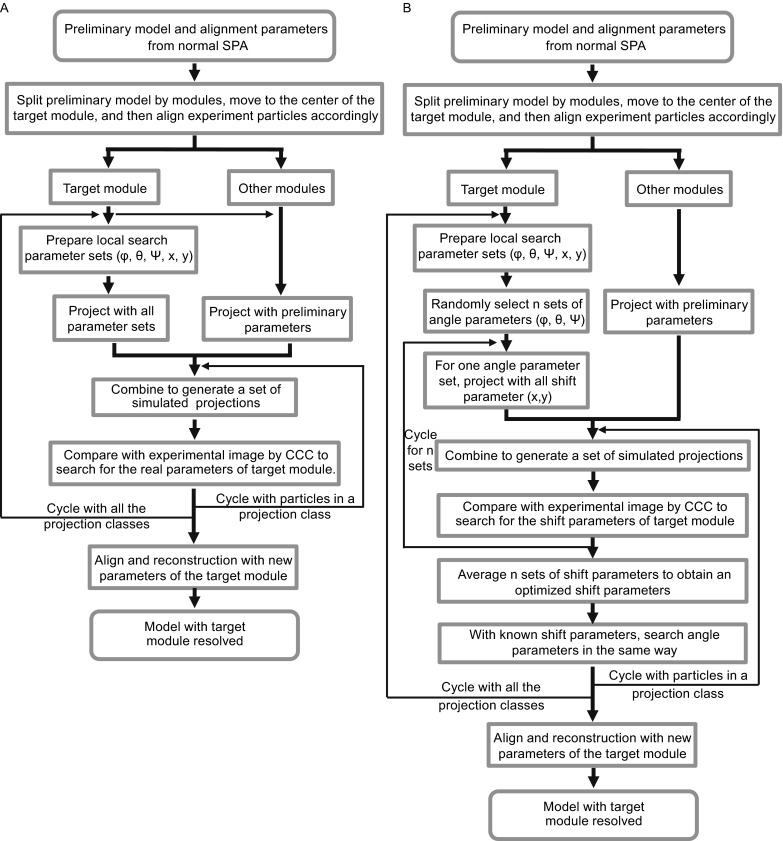
Diagram showing the local optimization procedures with two different strategies. (A) The procedure for searching the shift (x, y) and angle parameters (φ, θ, ψ) simultaneously and exhaustively. (B) The procedure for searching the shift (x, y) and angle parameters (φ, θ, ψ) separately

We propose the following procedure to optimize the orientation of the target module (Figs. 1, 3). Firstly, the preliminary model reconstructed from the conventional SPA procedure is divided into two parts, the target module to be optimized and the remaining region, by using a pre-determined mask. Then the entire model is shifted so that the center of the target module is placed at the center of the volume/box. The parameters (φ, θ, ψ, x, y) of all experimental particles from the conventional SPA procedure are transformed according to the model shift, to make all particle aligned with the shifted model, thereby yielding the preliminary parameters of the target module for every experimental image. The reason for placing the target module at the origin is to keep the in-plane x-y shift search independent from the orientation angles search. This has the advantage of saving computation resources. In addition, 3D reconstruction of the target module can be improved due to a better tolerance for angular parameter errors at the center area (Zhang and Ren, 2012).

With the preliminary parameters for each particle, all of possible parameters defined by (11), (15) and (16) are considered for the target module. The target module is transformed with all possible orientation parameters (φ, θ, ψ, x, y) and then combined together with the rest part of the model to generate a series of simulated projections, which are compared with the experimental image by using CCC defined by (1). The optimized orientation parameters of the target module for each particle are determined according to the highest CCC. All of the operations (rotations and shift/translations) will be combined to minimize interpolation errors. With the optimized parameters for target module, a new and improved 3D reconstruction can be generated using the conventional 3D reconstruction method (e.g. WBP, SIRT or Fourier method).

Here we develop two procedures to search the optimized parameters of the target module. The first one is to perform an exhaustive search of angle parameters (φ, θ, ψ) and shift parameters (x, y) simultaneously, which requires to generate simulating images with every combination of angle and shift parameters to make comparison (Fig. 3A). The second procedure is to separate shift parameter searching from angle parameter searching (Fig. 3B). We randomly choose n sets of angle parameters within the constriction defined by (11) and (15) and then search all possible shift parameters defined by (16). As a result, n sets of optimized shift parameters are obtained, which are averaged to reduce error. Thereafter, an exhaust search of the angle parameters within the defined ranges is performed with the pre-optimized shift parameters.

## RESULTS

**Figure 4:**
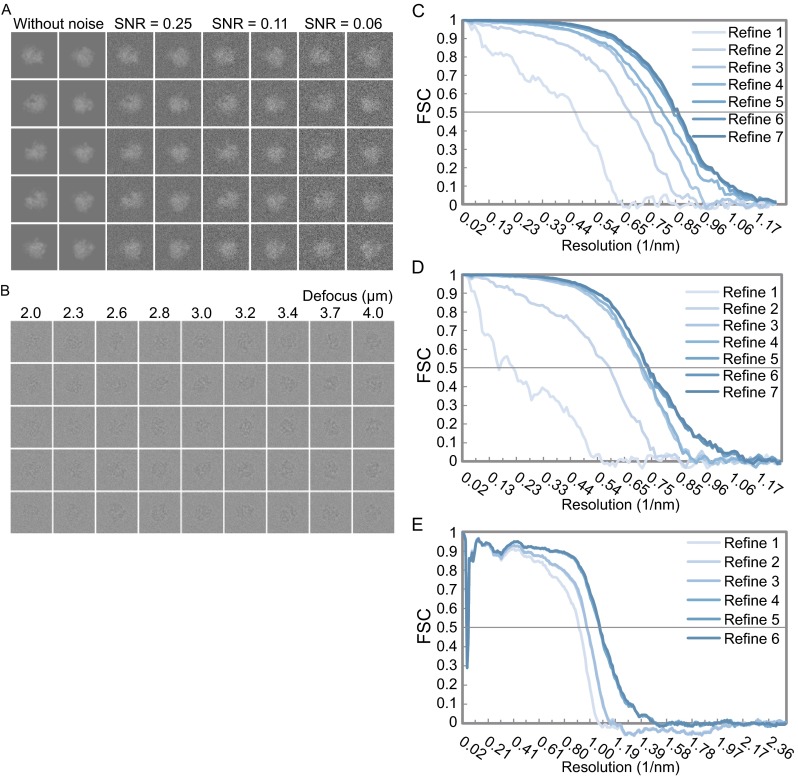
Simulated datasets and their FSC convergences during reconstruction refinements by conventional SPA procedure. (A) Every two columns represent the simulated projections of artificial ribosome with Gaussian noise added in different SNR levels. The left two columns represent the original projections. (B) Simulated projections of artificial ribosome generated from InSilicoTEM (Vulovic et al., 2013) in different defocus from −2.0 μm to −4.0 μm. (C), (D) and (E) The FSC curves of those simulated datasets ((C) is for the dataset in (A) with SNR of 0.25, (D) for the one in (A) with SNR of 0.11 and (E) for the one in (B)) during reconstruction refinement iterations by conventional SPA procedure. The FSC was calculated between the reconstructed map and the ground-truth map generated from PDB files (PDB code 4V7H for the datasets with Gaussian noise in (A), and PDB codes 4V7C for the dataset generated from InSilicoTEM in (B)). The final assessed resolutions at FSC = 0.5 by the conventional SPA procedure are indicated and also shown in Table 1

To test our LO-refinement algorithm, we generated two datasets, using the 80S and 70S ribosomes as the test samples. The relative orientation and position between the large subunit and the small subunit are randomly varied within a small range. The first dataset contains projections of ribosomes (80S, PDB code: 4V7H) with various levels of Gaussian noises added to yield signal-to-noise ratios (SNR) of 0.25, 0.11 and 0.06 (Fig. 4A). The second one contains the ribosome (70S, PDB code: 4V7C) projections that are generated by using the electron microscopy simulation software InSilicoTEM (Vulovic et al., 2013), with the effects of contrast transfer function (CTF) and camera taken into account (Fig. 4B).

### Reconstruction and LO-refinement for the datasets with Gaussian noise

With the conventional SPA procedure, the 3D density map of the 80S ribosome was reconstructed from the datasets with Gaussian noise. The resolution was assessed at FSC = 0.5 by calculating the FSC curve between the reconstructed map and the ground-truth map generated from the PDB file. For the dataset with SNR of 0.25, the conventional SPA procedure produced a final reconstruction with the resolution of 11.5 Å (Fig. 4C). For the dataset with SNR of 0.11, the resolution of the final density map was assessed to be at 13.5 Å (Fig. 4D). However, for the dataset with SNR of 0.06, our conventional SPA procedure could not yield a good reconstruction due to the extremely low SNR. As a result, in the following LO-refinement procedure, only the datasets with SNR of 0.25 and 0.11 were tested.

**Figure 5:**
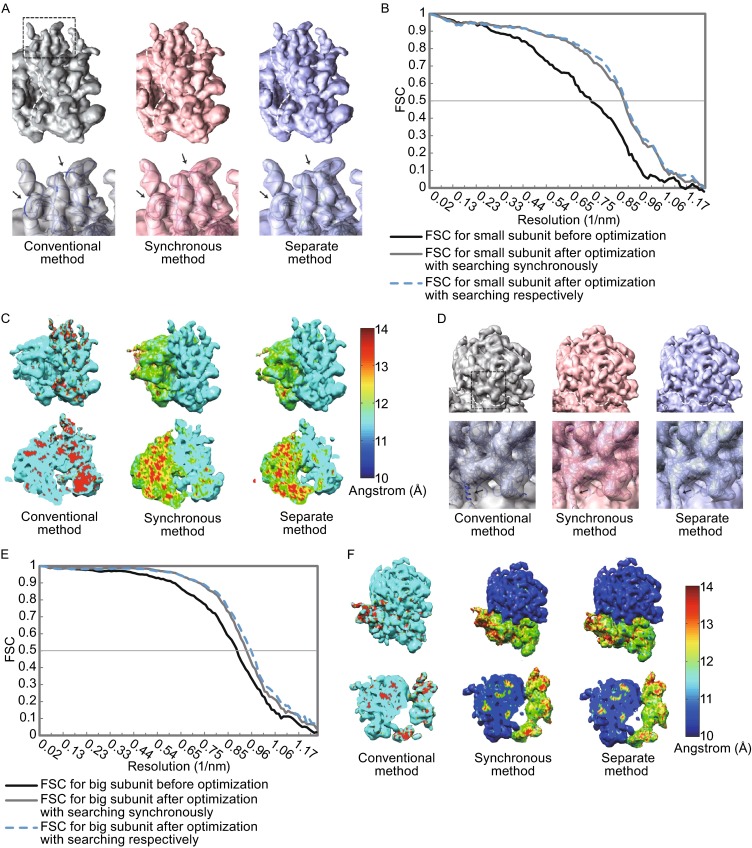
The improvement by the LO-refinement procedure for the dataset with Gaussian noise of SNR = 0.25. All the density maps for comparison are shown in the same threshold. (A) Comparison of the small subunit maps reconstructed from conventional SPA procedure (left in grey), LO-refinement procedure with the simultaneous parameter-searching strategy (middle in red, see also Fig. 3A) and the LO-refinement procedure with the separate parameter-searching strategy (right in blue, see also Fig. 3B). Top, 3D density maps of small subunits and the edges between small and large subunits are depicted with white dashed lines. Bottom, a zoom-in view of the reconstructed small subunit at the area indicated with the black dashed lines on the top. The maps are corrected with EM-BFACTOR (Fernandez et al., 2008) to 11.1 Å for emphasizing the information near the target resolution and then fitted with the crystal structures. The improvements of the density quality after LO-refinement are indicated with black arrows. (B) The FSC curves between the reconstructed map of the small subunit and the ground-truth map generated from the PDB file (PDB entry 4V7H). (C) Local resolution analysis of the reconstructed density map. The map (up row) is colored according to the corresponded local resolution that is computed by ResMap (Kucukelbir et al., 2014). One representative slice of the map with local resolution colored is shown below accordingly. (D) Comparison of the large subunit maps reconstructed from conventional SPA procedure (left in grey), LO-refinement procedure with the simultaneous parameter-searching strategy (middle in red, see also Fig. 3A) and the LO-refinement procedure with the separate parameter-searching strategy (right in blue, see also Fig. 3B). Top, 3D density maps of large subunits and the edges between large and small subunits are depicted with white dashed lines. Bottom, a zoom-in view of the reconstructed large subunit at the area indicated with the black dashed lines on the top. The maps are corrected with EM-BFACTOR (Fernandez et al., 2008) to 10.4 Å for emphasizing the information near the target resolution and then fitted with the crystal structures. The improvements of the density quality after LO-refinement are indicated with black arrows. (E) The FSC curves between the reconstructed map of the large subunit and the ground-truth map generated from the PDB file (PDB entry 4V7H). The assessed resolutions at FSC = 0.5 by different procedures are indicated and also shown in Table 1. (F) Local resolution analysis of the reconstructed density map with the same scheme in (C)

For the small subunit in the dataset of SNR 0.25,the LO-refinement method yielded a less-noisy density map that fits better with the ground-truth structure in comparison with the reconstruction from the conventional SPA procedure (Fig. 5A). The LO-refinement also improved the resolution (FSC = 0.5) from 13.4 Å to 11.2 Å for the exhaustive searching strategy and from 13.4 Å to 11.1 Å for the separate searching strategy (Fig. 5B and Table 1). The improvement by LO-refinement method was further analyzed and confirmed by ResMap (Kucukelbir et al., 2014) that computes the local resolution of the reconstructed map. It is clear to observe that the local resolution of the small subunit is significantly improved after LO-refinement while that of the large subunit is compensated as we predicted (Fig. 5C).

**Table 1:** Assessed resolutions at FSC = 0.5 of reconstructions from three simulated datasets by conventional SPA procedures and LO-refinement procedures

Dataset	Subunit	Conventional SPA reconstruction before LO-refinement	LO-refinement by searching shifts and angles simultaneously	LO-refinement by searching shifts and angles separately
With Gaussian noise at SNR = 0.25	Small	13.4 Å	11.2 Å	11.1 Å
	Large	11.1 Å	10.6 Å	10.4 Å
With Gaussian noise at SNR = 0.11	Small	15.5 Å	12.8 Å	12.2 Å
	Large	13.1 Å	11.7 Å	11.6 Å
Generated from InSilicoTEM	Small	9.7 Å	9.1 Å	8.9 Å
	Large	9.1 Å	8.9 Å	8.7 Å

For the large subunit in the dataset of SNR 0.25,the LO-refinement method also yielded a less-noisy density map with a better fit to the ground-truth structure (Fig. 5D) and improved the resolution (FSC = 0.5) from 11.1 Å to 10.6 Å for the exhaustive searching strategy and from 11.1 Å to 10.4 Å for the separate searching strategy (Fig. 5E and Table 1), which is further proved by local resolution analysis using ResMap (Kucukelbir et al., 2014).

**Figure 6:**
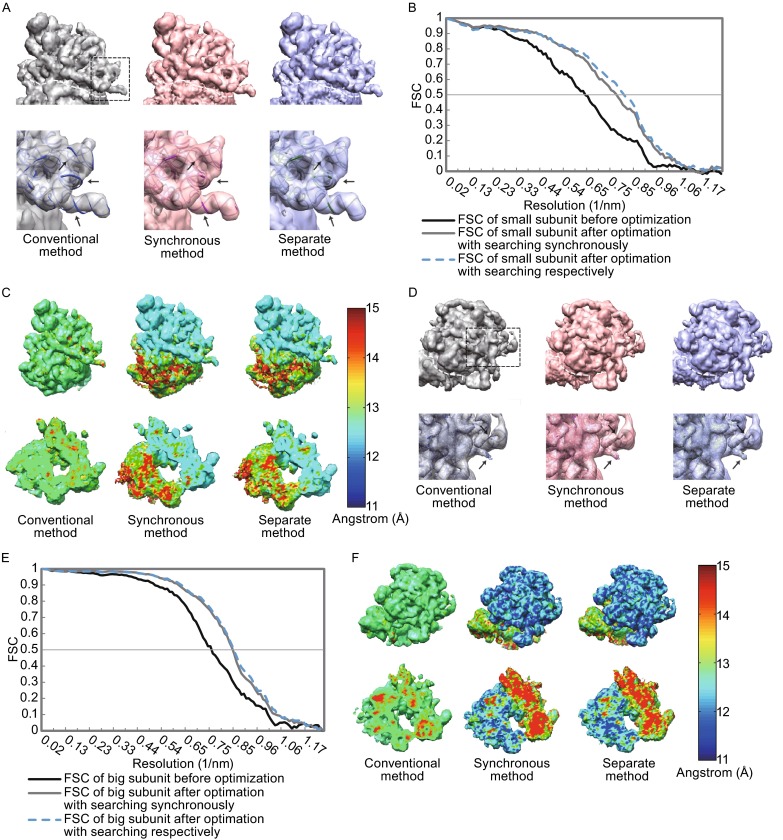
The improvement by the LO-refinement procedure for the dataset with Gaussian noise of SNR = 0.11. The scenario is same as that in Fig. 5. In brief, (A) and (D) are the comparisons of reconstructions for small subunits (A) and large subunits (D). (B) and (E) are the corresponding FSC curves respectively. (C) and (F) are the local resolution analyzes of the reconstructed density maps. For detailed descriptions, see Fig. 5

For the dataset of SNR 0.11, we also observed a significant improvement by using the LO-refinement method. The resolution (FSC = 0.5) of the small subunit was improved from 15.5 Å to 12.8 Å for the exhaustive searching strategy and from 15.5 Å to 12.2 Å for the separate searching strategy (Fig. 6A–C and Table 1). And the resolution (FSC = 0.5) of the large subunit was improved from 13.1 Å to 11.7 Å for the exhaustive searching strategy and from 13.1 Å to 11.6 Å for the separate searching strategy (Fig. 6D–E and Table 1).

We observed that, after LO-refinement, the quality of the density map and the assessed resolution at FSC = 0.5 for the target subunit were significantly improved while those for the non-target subunit became worse (Figs. 5C, 5F, 6C and 6F). The reason for this observation is that the LO-refinement procedure increases the accuracy of the parameters for the target module and thereby at the same time the accuracy of those for the non-target module is decreased due to the varied relative position and orientation between the target (refined) and non-target modules (not refined).

We also observed that, LO-refinement improves the reconstruction more significantly for the small subunit than for the large subunit (Table 1). This is likely due to that the large subunit has more weight to contribute to the final projection, leading to a smaller error of orientation determination. As a consequence, the room for improvement in the accuracy of orientation determination is smaller for the large subunit than for the small subunit.

Furthermore, we found that the two different optimization strategies yield different reconstruction resolutions for the same target module (Table 1). The separate searching strategy (Fig. 3B) had a slightly better result than the exhaustive simultaneous searching strategy (Fig. 3A). One reason for this is that for the separate searching strategy the final shift parameters are the average of ten optimized values from ten randomly selected trial angles, which overcomes the limitation of sampling only in integer steps, thereby increasing the accuracy of shift parameter determination. It is worth noting that, the separate searching strategy is also more efficient, requiring less intensive computation than that of the exhaustive simultaneous searching strategy.

### Reconstruction and optimization for the datasets generated by InSilicoTEM

**Figure 7:**
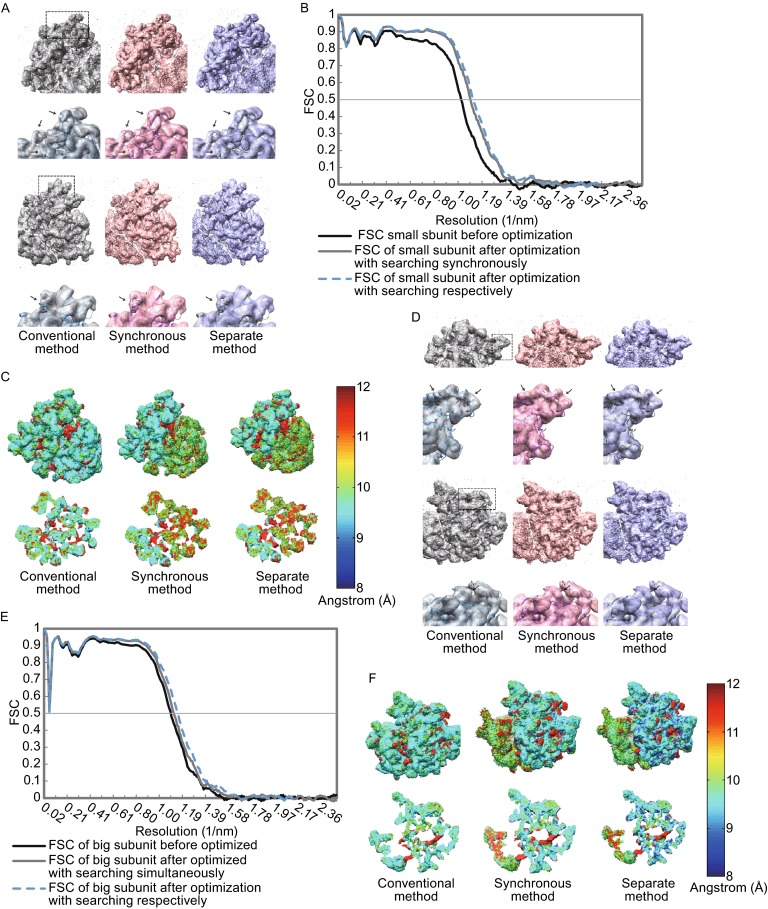
The improvement by the LO-refinement procedure for the dataset generated by InSilicoTEM. The scenario is same as that in Fig. 5. In brief, (A) and (D) are the comparisons of reconstructions in different views for small subunits (A) and large subunits (D). (B) and (E) are the corresponding FSC curves respectively. (C) and (F) are the local resolution analyzes of the reconstructed density maps. Differently, the ground-truth structures here are from another PDB file (PDB entry 3J5T) for the small subunit (A and B) and the one (PDB entry 3J5U) for the large subunit (D and E). The maps at the second and fourth row in (A) and (D) are corrected with EM-BFACTOR (Fernandez et al., 2008) to 8.9 Å and 8.7 Å respectively for emphasizing the information near the target resolution. The significant fluctuations in the low frequency part of FSC curves in (B) and (E) are due to the oscillation of contrast transfer function. For detailed descriptions, see Fig. 5

For the 58,542 particles generated by InSilicoTEM, the conventional SPA procedure yielded a final reconstruction with a resolution of 9.3 Å (Fig. 4E). Further LO-refinement yielded a less-noisy density map with a better fit to the ground-truth structure in comparison with the reconstruction from the conventional SPA procedure for both the small and the large subunits (Fig. 7A and 7D). The resolution (FSC = 0.5) of the small subunit was improved from 9.7 Å to 9.1 Å for the exhaustive searching strategy and from 9.7 Å to 8.9 Å for the separate searching strategy (Fig. 7A–C and Table 1). And the resolution (FSC = 0.5) of the large subunit was improved from 9.1 Å to 8.9 Å for the exhaustive searching strategy and from 9.1 Å to 8.7 Å for the separate searching strategy (Fig. 7D–E and Table 1). It should be noted that, the apparent inconsistency between all the reconstructed maps and the ground-truth structure in low frequency (Fig. 7B and 7E) is likely due to the insufficient defocus groups during dataset generation using InSilicoTEM.

In addition, similar to the above datasets with Gaussian noise, besides a better reconstruction resolution, the separate searching strategy here is also faster than the exhaustive simultaneous searching strategy (Fig. 7C and 7F, Table 2).

**Table 2:** Computation consumptions during optimizing parameters of the InSilicoTEM generated dataset for the two parameter-searching strategies

	Simultaneous search strategy	Separate search strategy
Node configuration (CPU type, MHZ, cache size, memory)	Intel Xeon X5650, 2.67 GHz, 12 MB, 36 GB	Intel Xeon X5650, 2.67 GHz, 12 MB, 36 GB
Number of processors per node	12	12
Number of nodes	7	7
Network (I/O)	1GB Ethernet	1GB Ethernet
Storage	NFS Disk array (SATA II, 7200 rpm, raid5)	NFS Disk array (SATA II, 7200 rpm, raid5)
Number of particles	58542	58542
Size of particle	128 × 128	128 × 128
Number of projections	5000	5000
Computation time of LO-refinement for the small subunit	36.7 h	7.8 h
Computation time of LO-refinement for the large subunit	37.6 h	8.1 h

All of above, for the dataset close to experimental electron microscopic conditions with CTF modulation and camera effect, the LO-refinement algorithm can still work effectively to improve the map quality and the reconstruction resolution of the target module.

## DISCUSSION

The conventional method in single particle analysis of heterogeneous sample with multiple conformations is to perform 2D or 3D classifications to try to separate different conformations into independent classes. The success of the conventional method is based on the assumption that the target macromolecular complex could only exhibit a small number of conformations. However, this assumption is challenged by the fact that many macromolecular complexes behave in a dynamic equilibrium with continuous conformational changes.

The work presented herein describes an image-processing algorithm, named as local optimization refinement (LO-refinement), to improve the reconstruction quality and resolution in single particle analysis of macromolecular complexes with infinite conformations. The assumption of LO-refinement is that the macromolecular complex can be treated as a combination of multiple modules, with each module exhibiting a relatively rigid conformation within our interested resolution. And the multiple conformations of the complex can be regarded as slightly varied relative positions and orientations among different rigid modules. Although the assumption is demanded, we realize that it reflects the nature of many macromolecular machines and is applicable to a large number of cases.

The main idea of the LO-refinement procedure is to focus on each rigid module and optimize its orientation and position individually. By maximizing the cross correlation coefficient (CCC) between the experimental projection and a series of simulated projections that are comparable to the experimental projection with varied orientation and position of the target module, we could obtain optimized parameters to improve the reconstruction of the target module. During the calculation of CCC, we apply a mask around the target module to reduce the contribution from the non-target modules, thereby increasing the accuracy of parameter determination for the target module. For parameters searching, we used two strategies, the exhaustive search for both angles and shifts and the separate search for angles and shifts, which is similar to the previously reported image alignment algorithms (Joyeux and Penczek, 2002). For the reconstruction step, we move the focused part to the center of the volume where the resolution is always higher than the surrounding since a better tolerance for the errors of angular parameters.

For a proof of principle of our LO-refinement procedure, we generated two types of datasets using the structures of ribosomes with two relative rigid modules (large and small subunits). One dataset incorporated Gaussian noises of different levels into the projections. The other dataset was generated using the program InSillicoTEM (Vulovic et al., 2013) to incorporate near experimental microscopic effects including contrast transfer function and detective quantum efficiency of camera. Testing the LO-refinement procedure against both types of datasets showed significant improvements on both the map quality and the assessed resolution.

Besides the ribosome molecules with two assumed rigid modules, this LO-refinement procedure could in principle be applicable to the complexes with multiple modules. In these cases, the non-target modules can be treated as one integral part with their parameters of orientations and positions unchanged. With this procedure, all the modules of the complex can be reconstructed into a better resolution. Furthermore, it is clear that this LO-refinement procedure can be iterated to further optimize the parameters of all the target modules and improve the resolutions of their reconstructions.

It should be pointed out that our LO-refinement procedure requires the modules of a complex in rigid conformations within a specific resolution. In reality, various degrees of conformational variations likely exist for any given module, which can be further affected by the changes between modules, especially at higher resolutions. This may limit the improvement by this LO-refinement procedure to achieve atomic resolution in some cases. Nonetheless, in many cases the rigid module assumption is valid to atomic resolutions, from extensive experience learned from the practice of multi-domain/module non-crystallography symmetry (NCS) averaging in X-ray crystallographic studies. The challenge may be the appropriate identification of the rigid modules, which could be facilitated by the recently reported normal mode analysis method (Jin et al., 2014) that can analyze the internal conformational flexibility of a target module and a new analytical approach for determining the free-energy landscape and the continuous trajectories of molecular machines (Dashti et al., 2014). In addition, another challenge is to clearly define the interaction interfaces among different modules, which may not be resolved by this LO-refinement procedure.

This LO-refinement procedure could be further improved in the following aspects. First, the assumption of that both the terms (1) and (5) can reach maxima simultaneously at the same orientation of target module may not be valid in many cases. The inference of other non-target modules, term (8), during calculating CCC should be avoided in the next improvement. One solution to this problem is to remove the information of non-target modules from the experimental particle image. Besides, the real experimental particle image involves CTF (contrast transfer function) modulation of the particle projection while the CTF effect in the simulated model projection image has been corrected. One could not compare the experimental image and simulated image directly without considering the CTF effect. Thus, the theory of LO-refinement from term (1) to term (8) can be adapted and improved as follows.

The experimental image of particle projection with two modules A and B can be described as,

17 $$ f_{1} \left( {{r}_{{j}} } \right) = p_{1A} \left( {{r}_{{j}} } \right) \otimes PSF + p_{1B} \left( {{r}_{{j}} } \right) \otimes PSF $$

Where, $p_{1A} \left( {{r}_{{j}} } \right)$ and $p_{1B} \left( {{r}_{{j}} } \right)$ are the projections of module A and B respectively, PSF is the Fourier transform of CTF and $\otimes$ represents convolution.

The projected image of particle model with two modules A (target module) and B (non-target module) can be described as,

18 $$ p_{2} \left( {{r}_{{j}} } \right) = p_{2A} \left( {{r}_{{j}} } \right) + p_{2B} \left( {{r}_{{j}} } \right) $$

Where $p_{2A} \left( {{r}_{{j}} } \right)$ is the projection of module A with adjusted position and orientation and $p_{2B} \left( {{r}_{{j}} } \right)$ is the projection of module B with its preliminarily determined parameters of orientation and shift. Both $p_{2A} \left( {{r}_{{j}} } \right)$ and $p_{2B} \left( {{r}_{{j}} } \right)$ can be explicitly computed from the 3D module of the particle. There is no CTF modulation in term (18).

The information of module B in term (17) can be removed by minus a CTF modulated projection of module B.

19 $$ f_{1A} \left( {{r}_{{j}} } \right) = p_{1A} \left( {{r}_{{j}} } \right) \otimes PSF + p_{1B} \left( {{r}_{{j}} } \right) \otimes PSF - k*p_{2B} \left( {{r}_{{j}} } \right) \otimes PSF $$

Since the parameters of orientation and shift for module B are preliminarily determined in certain accuracy, $p_{1B} \left( {{r}_{{j}} } \right)$ and $p_{2B} \left( {{r}_{{j}} } \right)$ are roughly similar and the term

20 $$ \Delta_{B} = p_{1B} \left( {{r}_{{j}} } \right) \otimes PSF - k*p_{2B} \left( {{r}_{{j}} } \right) \otimes PSF $$

can be minimized close to zero by selecting appropriate scaling factor *k*.

Thus, the target CCC between experimental and simulated data can be written as

21 $$ \rho^{A}_{12} = \frac{{\mathop \sum \nolimits_{j = 1}^{J} \left[ {f_{1A} \left( {r_{j} } \right) - {<}f_{1A}\!\!{>}}\!\! \right]\left[ {f_{2A} \left( {r_{j} } \right) - {<}f_{2A}\!\!{>}}\!\! \right]}}{{\left\{ {\mathop \sum \nolimits_{j = 1}^{J} [f_{1A} \left( {r_{j} } \right) - {<}f_{1A}\!\!{>}\!\!]^{2} \mathop \sum \nolimits_{j = 1}^{J} [f_{2A} \left( {r_{j} } \right) - {<}f_{2A}\!\!{>}\!\!]^{2} } \right\}^{1/2} }} $$

Where $f_{1A} \left( {r_{j} } \right)$ is defined in term (19) and

22 $$ f_{2A} \left( {r_{j} } \right) = p_{2A} \left( {{r}_{{j}} } \right) \otimes PSF $$

Considering terms (19), (20) and (22), the numerator of $\rho^{A}_{12}$ in term (21) can be further written as

23 $$ \mathop \sum \limits_{j = 1}^{J} \left[ {p_{1A} \left( {{r}_{{j}} } \right) \otimes PSF - {<}p_{1A} \left( {{r}_{{j}} } \right) \otimes PSF\!\!{>}}\!\!\right]\left[ {p_{2A} \left( {{r}_{{j}} } \right) \otimes PSF - {<}p_{2A} \left( {{r}_{{j}} }\right) \otimes PSF\!\!{>}}\!\! \right] $$

24 $$ + \mathop \sum \limits_{j = 1}^{J} \left[ {\Delta_{B} - {<}\Delta_{B}\!\!{>}}\!\! \right]\left[ {p_{2A} \left( {{r}_{{j}} } \right) \otimes PSF - {<}p_{2A} \left( {{r}_{{j}} } \right) \otimes PSF\!\!{>}}\!\! \right] $$

Where term (24) is close to zero and term (23) can reach the maxima together with the correlation between $p_{1A} \left( {{r}_{{j}} } \right)$ and $p_{2B} \left( {{r}_{{j}} } \right)$,

25 $$ \mathop \sum \limits_{j = 1}^{J} \left[ {p_{1A} \left( {{r}_{{j}} } \right) - {<}p_{1A} \left( {{r}_{{j}} } \right)\!\!{>}}\!\! \right]\left[ {p_{2A} \left( {{r}_{{j}} } \right) - {<}p_{2A} \left( {{r}_{{j}} } \right)\!\!{>}}\!\! \right] $$

As the result, the target CCC defined in term (21) can reach the maxima only if the cross-correlation of projections between experimental and simulated module A defined in term (25) reaches the maxima. This improved theory of LO-refinement described from term (17) to term (25) can fully avoid the inference of non-target modules and account the effect of CTF modulation, and thereby would yield further improved reconstruction of the target modules, especially when dealing with the real experimental data.

In addition, besides back projection, other reconstruction algorithms, i.e. SIRT (Bangliang et al., 2000) and NUFFT (Chen and Förster, 2014), can be applied. Furthermore, maximum likelihood probability (Dempster et al., 1977) and Bayesian analysis (Scheres, 2012a) could also be implemented in this LO-refinement procedure.

During the revision of the present paper, we noticed that the recent publication by Liu and Cheng (2015), where they developed an image processing method to reconstruct the high-resolution map of viral internal structure within the capsid, described the detailed math of how to subtract the information of viral internal structure from the raw experimental whole virus particle. The idea of their information subtraction is similar to our proposed adjusted LO-refinement theory in this discussion from term (17) to term (25).

Furthermore, we also noticed that Nguyen et al. recently reported the cryoEM structure of pre-assembled spliceosomal complex and they developed an image processing approach called “multi-body refinement” to improve the density for the flexible arm domain (Nguyen et al., 2015). The idea of their “multi-body refinement” is similar to our LO-refinement but implemented differently in Fourier space and combined together with Bayesian approach. The success of their “multi-body refinement” approach has become another proof of the idea described in this paper. By implementing the adapted LO-refinement theory with improved codes for efficient computation, our LO-refinement algorithm will provide an alternative solution in real space to deal with the conformational flexibility of macromolecular complexes for single particle analysis.

## MATERIALS AND METHODS

### Test the datasets with Gaussian noise

A 3D map of the 80S ribosome (Taylor et al., 2009) was generated from PDB file (PDB entry 4V7H) by using the command e2pdb2mrc.py in EMAN2 (Ludtke et al., 1999) with a pixel size of 4 Å. Subsequently, a rotation around an axis through the subunit center and a shift in 3D space were applied to each subunit independently using the commands CG, ROT L and SH in SPIDER (Frank et al., 1996).

For simulating the scenario of slight and random flexibility between the large and small subunits, the direction of the rotational axis was selected randomly, and the rotational angle was assigned randomly in a normal distribution with an average value of 0° and a standard deviation of 1.67°. The shifts x, y, z were also assigned randomly in a normal distribution with an average value of 0 pixel and a standard deviation of 1 pixel. The two randomly moved subunits were then combined together to generate a whole 80S molecule. In total, 50,000 density maps were generated in this way and each map was projected once with the projection direction randomly selected. As a result, we simulated a dataset of a molecular complex with multiple conformations that are fixed in ice with random orientations.

In the final step of generating the simulation data, we added Gaussian noise into each projection by using the commands FS, MO and ADD in SPIDER (Frank et al., 1996). Three different (0.25, 0.11 and 0.06) SNR of noises were used according to previous studies (Baxter et al., 2009), yielding three datasets with different levels of noises (Fig. 4A).

To carry out 3D reconstruction, we first applied a conventional SPA routine using a customized SPIDER script in the Liu lab (Huang et al., 2012) to perform reconstruction refinement against the above simulated datasets. The density map of the whole ribosome that was generated from the corresponding PDB file was low-pass filtered to 20-Å resolution as an initial model. For each cycle of the refinement, the FSC (Fourier shell correlation) curve between the refined map and the PDB-generated density map was calculated by using the command FSC in SPIDER.

After the conventional SPA refinement became converged (Fig. 4C and 4D), we applied the LO-refinement to both the small and large subunits respectively. Both procedures of the LO-refinement described above (Fig. 3) were tested. The subunits were segmented using Chimera (Pettersen et al., 2004). A round mask with a diameter of 30 pixels for the small subunit, or 40 pixels for the large subunit was used during CCC computation to select the target module while excluding the background noise and the signal from the non-target module. The projection direction of the target module was searched within a cone of 10° and the in-plane shift was searched within a range from −4 to 4 pixels. For the second optimization strategy (Fig. 3B), the number of randomly selected angles was set to 10. To ensure that the improvement by using this LO-refinement was not due to increased sampling rate, both the angular and the shift sampling steps were kept the same (2° and 1 pixel respectively) as the last cycle of refinement in the conventional SPA procedure. Similarly, the 3D reconstruction methods were also kept the same as the weighted back projection (WBP) that was carried out using the command BP 32F in SPIDER.

After one iteration of LO-refinement, the density of the target module was segmented using a soft mask in the shape of the module, and the FSC curve was calculated against the density map generated from PDB file for comparison and further analysis (Scheres and Chen, 2012).

### Test the dataset generated from InSilicoTEM

We further validated our LO-refinement by using a simulated dataset with experimental conditions considered. We used the software package InSilicoTEM (Vulovic et al., 2013) to generate a new dataset of ribosome projections. This procedure takes into account the most relevant physical parameters of cryo-electron microscopy including both contrast transfer function and camera factors.

In this test, the coordinates of the 70S ribosome subunits (PDB entry 4V7C) (Brilot et al., 2013) were rotated and shifted respectively in the same way described above for the Gaussian type dataset on the 80S ribosome. The randomly moved subunits were then combined into a whole structure of the 70S ribosome.

In total, 58,542 ribosome structures were generated and each of them represents a slightly different conformation. Thereafter, the generated coordinates were submitted to InSilicoTEM for projection generation using the condition of 200 kV acceleration voltage and 2 Å/pixel in a CCD camera. 58,542 projections in 9 different defocus groups were generated, with each projection corresponding to a random conformation of ribosome in a random orientation. The parameters of InSilicoTEM are summarized in Table 3 and the representative projections generated from InSilicoTEM are shown in Fig. 4B.

**Table 3:** All the physical parameters used in InSilicoTEM to generate a simulated dataset

Specimen					
Motion blur	0 Å	Thickness of the specimen	38 × 10^−9^ m	Amplitude contrast	Plasmon of ice and protein
Electron-specimen interaction					
Interaction type	Weak phase				
Microscope					
Spherical aberration	2.7 × 10^−3^ m	Illumination aperture	0.030 × 10^−3^ rad	Chromatic aberration	2.7 × 10^−3^ m
Energy spread of the source		0.7 eV			
Aperture					
Diameter of objective aperture		100 × 10^−6^ m	Focal distance	4.7 × 10^−3^ m	
Acquisition settings					
Pixel size	0.2 × 10^−9^ m	Defocus	2.0, 2.3, 2.6, 2.8, 3.0, 3.2, 3.4, 3.7, 4.0 μm	Astigmatism	0 m
Acceleration voltage		200 kV		Dose rate	20 e-/Å^2^
Detector-camera					
Camera type		Gatan US4000	DQE	With DQE	

CTF correction (phase flipping) of the dataset generated from InSilicoTEM was performed using the command e2ctf.py in EMAN2. Then a conventional SPA procedure (Fig. 4E) and subsequent LO-refinement for each subunit were performed in the same way as described above for the Gaussian type dataset. Slightly differently, a binning factor of 2 was used for two-dimensional image alignment to reduce the computation time, while the reconstruction was calculated without binning. During LO-refinement, the angle and shift sampling steps (2° and 1 pixel respectively) and the reconstruction method (WBP using BP 32F in SPIDER) were kept the same as those in the last cycle of refinement in the conventional SPA procedure.

After one iteration of LO-refinement, the density of the target module was segmented using a soft mask in the shape of the module, and the FSC curve was calculated against the density map from PDB file for comparison and further analysis (Scheres and Chen, 2012). All the reconstructed maps were analyzed by ResMap (Kucukelbir et al., 2014).

## DATA ACCESSION

All the simulated datasets together with the final reconstructed maps are available at http://feilab.ibp.ac.cn/shared in case that those interested readers want to test their algorithms for comparison.
